# Optimization of Catalyst Synthesis Using Ornamental Stone Waste Supported on Activated Carbon for Enhancing Biodiesel Production Efficiency

**DOI:** 10.1021/acsomega.5c02065

**Published:** 2025-07-15

**Authors:** Fábio C. Aleixo, Diêgo N. Faria, Joycel V. Fernández, Daniel F. Cipriano, José G. A. Rodrigues, Gilberto M. Brito, Miguel A. Schettino, Amanda Bolsoni, Geisamanda P. Brandão, Leonardo L. L. Silveira, Jair C. C. Freitas

**Affiliations:** a Laboratory of Carbon and Ceramic Materials, Department of Physics, Federal University of Espírito Santo, Vitória, Espírito Santo 29075-910, Brazil; b Laboratory of Chemical Sciences, State University of the Northern Rio de Janeiro, Campos dos Goytacazes, Rio de Janeiro 28013-602, Brazil; c Engineering Unity, FAESA University Center − Vitória, Espírito Santo 29053-360, Brazil; d Atomic Spectrometry Laboratory, Department of Chemistry, Federal University of Espírito Santo, Vitória, Espírito Santo 29075-910, Brazil; e Center for Mineral Technology, Cachoeiro de Itapemirim, Espírito Santo 29300-970, Brazil

## Abstract

This work describes the optimization of the synthesis of carbon-supported heterogeneous catalysts containing CaO and MgO derived from ornamental stone waste (OSW) in view of their use for biodiesel production. The catalysts were prepared by mixing the OSW and coconut shell activated carbon (CSAC) powders with a NaOH solution under constant stirring; the mixture was then subjected to thermal treatments in an inert atmosphere. The variables of the synthesis process (thermal treatment temperature, residence time, and OSW/CSAC mass ratio) were analyzed using 2^3^ full factorial designs; the response variable was the biodiesel conversion, monitored by solution ^1^H nuclear magnetic resonance (NMR) spectroscopy. The presence of the phases CaO, MgO, Na_2_CO_3_, Na_2_Ca­(CO_3_)_2_, and Ca­(OH)_2_ (in amounts depending on the synthesis conditions) was identified by X-ray diffraction and solid-state ^23^Na NMR. The biodiesel conversion varied between 47 and 85%, with the highest value corresponding to the catalyst prepared using an OSW/CSAC mass ratio of 50:20 and thermally treated at 800 °C for 1 h. The main benefit of using the porous carbon support for the catalytically active phases was the significant reduction in the number of metals leached into the produced biodiesel.

## Graphical abstract

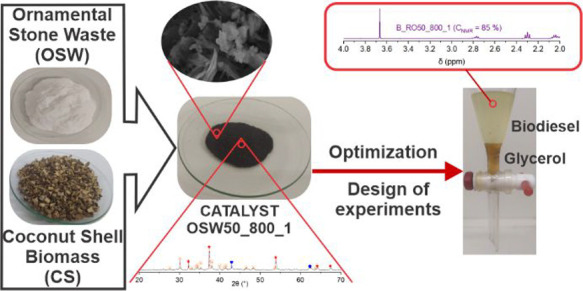

## 1 Introduction

Global energy demand is still highly dependent on fossil fuels.^1^ This dependence intensifies several environmental problems, such as greenhouse gas emissions generated by the burning of these fuels.^2^ In this context, the challenge arises of suppressing global energy demand and, at the same time, ensuring the well-being of future generations.^3^ Biodiesel has been considered a promising substitute for conventional diesel oil, since it is a biofuel of renewable origin and emits less CO, SO_2_ and hydrocarbons than petroleum-derived fuels.^4^ Thus, biodiesel appears on the world stage as an alternative and sustainable energy source capable of meeting part of the global energy demand.^5^

Transesterification has been routinely used to produce biodiesel because it provides high biodiesel conversion using short reaction times even at room temperature.^6^ In this process, biodiesel is produced by the reaction of triglycerides, such as the ones found in vegetable oils or animal fats, with short-chain alcohols, such as methanol or ethanol, in the presence of a catalyst.^4,7^

In general, homogeneous catalysts offer high activity and reaction speed, but they face significant challenges such as the generation of effluents and difficulty in reuse.^8^ To overcome these limitations, researchers have sought to develop heterogeneous catalysts by using different types of waste.^9^ The choice of heterogeneous catalysts is justified by their advantages, such as the simplicity of the purification and separation process, in addition to their reusability.^10^

Heterogeneous catalysis has emerged as a promising alternative for biodiesel production, particularly through the use of solid catalysts, which offer operational and environmental advantages over conventional homogeneous catalysts.^3,11,12^ Historically, the first studies involving solid catalysts for biodiesel synthesis date back to the late 1990s, when researchers began investigating metal oxides and zeolitic materials as viable alternatives to liquid alkaline catalysts.^3,11,12^ Since then, a wide range of solid catalysts has been developed, including materials derived from agro-industrial waste and catalysts supported on porous matrices, with the aim of enhancing efficiency and reducing costs.^3,13^ The main advantages of using solid catalysts include their easy separation from the reaction medium, lower waste generation, reusability, higher selectivity, and reduced corrosiveness compared to homogeneous systems. These factors contribute to more sustainable processes, with reduced environmental impact and greater economic feasibility at the industrial scale. Solid catalysts have been, for example, employed in the production of biodiesel from waste frying oil (WFO), which is widely used as a sustainable feedstock.^3,13^

Supported catalysts are produced for several reasons.^14^ The main role of the support is to promote the dispersion of the active phase, reducing its leaching when the catalyst is used.^15^ In addition, supported catalysts are generally more stable than conventional catalysts, besides being less susceptible to inactivation of the active sites and exhibitting improved selectivity.^14^ Carbonaceous materials are frequently used as support matrices for catalysts used in the production of biofuels. These materials have a relatively inert porous structure, which increases the metal–support interaction during the preparation of the catalyst, providing the ideal selectivity for the desired application. A carbonaceous matrix widely used as a support is activated carbon.^16^

The high cost is considered the biggest obstacle for the commercial production of biodiesel.^17^ The catalyst represents the second highest cost in biodiesel production, surpassed only by the raw material.^9^ Developing heterogeneous catalysts from waste materials can be a promising alternative to reduce both environmental impacts and production costs.^18^ Therefore, it is essential to develop and improve “green methodologies” for producing heterogeneous catalysts by using waste materials.^19^

Ornamental stone waste (OSW) is a rich source of valuable metal oxides such as CaO and MgO, making it a promising material for various catalytic applications.^20–22^ This waste material is a low-cost, accessible resource that requires no prior treatment, making it ideal for direct use in the preparation of catalysts useful for biodiesel production.^23^ Depending on the source, some of the main mineral constituents of OSW are calcite (CaCO_3_) and dolomite (CaMg­(CO_3_)_2_). Depending on the dolomite (mineral) content, carbonate stones can be divided into limestone (0–10%), dolomitic limestone (10–50%), calcitic dolomite (50–90%) and dolomite (90–100%).^24^ Although MgO generally lowers catalytic activity due to its lower basicity compared to CaO, its presence can enhance the stability of the catalyst, improving its resistance to deactivation throughout the catalytic process.^25^

Murguía-Ortiz et al.^26^ prepared sodium-doped dolomites through a two-step process. The material was calcined at 900 °C for 4 h and then mechanically mixed with sodium nitrate, followed by a second heat treatment at 900 °C for an additional period of 4 h. Yahaya et al.^27^ enhanced the catalytic activity of dolomite for biodiesel production by doping it with K_2_O. The catalyst preparation involved adding calcined dolomite, treated at 850 °C for 3 h, to a solution of K_2_CO_3_ in distilled water, followed by a second heat treatment under the same conditions. Widayat et al.^28^ developed a magnetic nanocatalyst from dolomite and iron sand. Fe_3_O_4_ was synthesized from iron sand using coprecipitation, while dolomite was calcined at 700 °C for 2 h to yield CaO/MgO; this product was then dissolved in distilled water and Fe_3_O_4_ was added to the solution. The resulting mixture was dried and subsequently heat-treated at 700, 850, and 1000 °C for 4 h to finalize the catalyst preparation.

In a recent work, Aleixo et al.^29^ studied methodologies for the synthesis of heterogeneous catalysts supported on activated carbon (AC), starting from OSW. In that study, they showed that the amounts of metals leached into the produced biodiesel was quite significant (374.5 mg/kg of Ca 12.6 mg/kg of Mg) when the material obtained after heat treatment of OSW was used as catalyst; on the other hand, when activated carbon was used as support, the leaching of Ca (<0.76 mg/kg) and Mg (<0.15 mg/kg) remained below the limits established by the European standard EN-14214.^30^

Considering that the possibility of reuse and the low cost of heterogeneous catalysts play a fundamental role in the economic and environmental viability of producing biodiesel,^31^ it is worth pursuing further investigations about the procedures for preparation of carbon-supported catalysts derived from waste materials. In this sense, the present work describes the optimization, using factorial planning, of the production of heterogeneous catalysts supported on AC, using OSW as a source of Ca and Mg oxides. The chosen support was an AC sample prepared from coconut shells by physical activation with steam and the catalysts were prepared by mixing the OSW and AC powders with a NaOH solution under constant stirring; the mixture was then subjected to thermal treatments in an inert atmosphere at different temperatures and during varied residence times. The variables of the production process (thermal treatment temperature, residence time, and OSW/AC mass ratio) were analyzed using 2^3^ full factorial design, considering the conversion of soybean oil into biodiesel as the response variable.

The present study aims to contribute to the development of sustainable catalytic systems by optimizing a green and cost-effective methodology for producing heterogeneous catalysts supported on activated carbon, using OSW as the precursor of active metal oxides. The main novelty of this work lies in the combined use of industrial residues (OSW and coconut shells) to obtain materials with technological interest, thus adding value to abundant wastes originated from different industrial sectors. Moreover, another innovative aspect of this work is the application of full factorial experimental design to systematically evaluate the influence of key process variables – namely, temperature, residence time, and OSW/AC mass ratio – on the performance of the prepared catalysts in the transesterification of soybean oil to obtain biodiesel. Unlike previous studies, this approach enables the preparation of supported catalysts that not only exhibit high catalytic activity but also significantly reduce metal leaching into the final biodiesel product, thereby addressing both performance and environmental compliance with international standards.

## 2 Experimental Section

### 2.1 Materials

The heterogeneous catalysts described in this work were prepared using OSW and coconut shells (CS) as starting materials. The OSW samples were collected from Cachoeiro de Itapemirim, in the state of Espírito Santo, in South-East Brazil, while CS samples were obtained from Vitória, Espírito Santo, Brazil. The coconut shells were converted into AC using physical activation with steam, as described elsewhere;^29^ this material is designated here as CSAC sample. Biodiesel was produced via the transesterification of vegetable oil, using commercial soybean oil (Soya brand) as the feedstock and methanol P.A. (CH_3_OH, Proquímios, 99.8%) as the reactant. The soybean oil used in this work exhibited a moisture content of 0.054 ± 0.002 wt % (determined by the Karl Fischer method)^32^ and the main fatty acids present in this material (determined by gas chromatography, see below) were methyl octadeca-9,12-dienoate, methyl octadec-6-enoate, and methyl hexadecanoate. ^1^H nuclear magnetic resonance (NMR) spectroscopy was used to quantify the biodiesel conversion, using deuterated chloroform (CDCl_3_, Sigma-Aldrich, 99.8% D) with 1% v/v tetramethylsilane (TMS) as the solvent. Anhydrous sodium carbonate P. A. (Na_2_CO_3_, minimum purity of 99.5%, Isofar; sum of impurities <0.04%).

### 2.2 Catalyst Precursors

The catalyst precursors were prepared by mixing OSW and CSAC powders with 50.0 mL of a NaOH solution (3.75 mol/L) in a Teflon beaker and stirring the mixture magnetically for 1 h. Afterward, 50 mL of distilled water were added and the system was kept under stirring for an additional period of 1 h. The mixture was then transferred to an oven at 100 °C to evaporate the water and fully dry the material.^7^ The masses of OSW to CSAC were varied, as shown in Table 1, whereas the mass of NaOH was adjusted to represent a fraction of 30 wt % of each mixture.

**Table 1:** Values of the Masses of the Reactants Used in the Synthesis of the Indicated Catalyst Precursors

precursor	OSW (g)	CSAC (g)	NaOH (g)	OSW:CSAC:NaOH (wt %)
OSW20	10.08	25.01	15.03	20:50:30
OSW35	17.49	17.48	14.90	35:35:30
OSW50	25.08	10.03	15.01	50:20:30

### 2.3 Optimization of the Preparation of the Catalysts

Initially, the screening stage was conducted to evaluate the significance of the variables related to catalyst preparation.^33^ The variables analyzed were: OSW/CSAC mass ratio (x_1_), calcination temperature (x_2_), and calcination time (x_3_). Regarding the mass ratio (x_1_), it is worth noting that the NaOH weight fraction was consistently set at 30 wt % throughout the screening process. A full factorial design (2^3^ + central point replicates) was employed, resulting in 11 experiments, as shown in Table 2. The response variable was the biodiesel conversion, determined from the ^1^H NMR spectra, with experiments performed in random order.^34^ Experiments 9, 10, and 11 are replicates performed under the same conditions.

**Table 2:** Matrix of Experiments Corresponding to the 2^3^ Full Factorial Design, with Definition of the Samples’ Nomenclature, Identification of the Variables, and Description of Their Actual and Coded Levels Used in the Screening Stage of the Preparation of the Catalysts

		variables		
exp	catalyst nomenclature	**OSW/CSAC mass ratio (wt %)**x_1_	**temperature (°C)** x_2_	**residence time (h)** x_3_
1	OSW20_600_1	20/50 (−1)	600 (−1)	1 (−1)
2	OSW50_600_1	50/20 (+1)	600 (−1)	1 (−1)
3	OSW20_800_1	20/50 (−1)	800 (+1)	1 (−1)
4	OSW50_800_1	50/20 (+1)	800 (+1)	1 (−1)
5	OSW20_600_3	20/50 (−1)	600 (−1)	3 (+1)
6	OSW50_600_3	50/20 (+1)	600 (−1)	3 (+1)
7	OSW20_800_3	20/50 (−1)	800 (+1)	3 (+1)
8	OSW50_800_3	50/20 (+1)	800 (+1)	3 (+1)
9	OSW35_700_2	35/35 (0)	700 (0)	2 (0)
10	OSW35_700_2	35/35 (0)	700 (0)	2 (0)
11	OSW35_700_2	35/35 (0)	700 (0)	2 (0)

### 2.4 Biodiesel Production

The catalytic activity of the prepared catalysts was evaluated in the transesterification reaction of vegetable oil with methanol, following conditions established in the literature.^7,35,36^ The mass ratio of methanol to soybean oil used in this study was 0.5:1, with 3% catalyst loading.

All the masses used in the reactions are shown in Table 3.

**Table 3:** Masses of Soybean Oil, Methanol and Catalyst Used to Obtain the Biodiesel Samples

sample	soybean oil (g)	methanol (g)	catalyst (g)
OSW20_600_1	10.0629	5.0078	0.3070
OSW50_600_1	10.0535	5.0200	0.3046
OSW20_800_1	10.0366	5.0561	0.3019
OSW50_800_1	10.0214	5.0428	0.3034
OSW20_600_3	10.0475	5.0886	0.3055
OSW50_600_3	10.0590	5.0279	0.3095
OSW20_800_3	10.0508	5.0221	0.3063
OSW50_800_3	10.0413	5.0879	0.3007
OSW35_700_2	10.0660	5.0610	0.3032
OSW35_700_2	10.0627	5.0531	0.3041
OSW35_700_2	10.0180	5.0110	0.3029
OSW50_800_1_r1c	30.0135	15.2123	1.5047
OSW50_800_1_r2c	26.7659	13.4107	1.3389
OSW50_800_1_r3c	22.2917	11.1587	1.1138

Initially, the soybean oil was preheated to 60 °C to accelerate the transesterification process. Meanwhile, the catalyst and methanol were mixed in a round-bottom flask and stirred magnetically for 25 min. The transesterification reaction was initiated by adding the preheated soybean oil to the catalyst-methanol mixture, with a reflux system attached to the flask to prevent methanol evaporation. The reaction proceeded at 60 °C under atmospheric pressure with continuous stirring for 3 h. Afterward, the catalyst was separated by centrifugation, and the resulting biodiesel was heated in a water bath at 80 °C for 1 h to remove the excess of methanol. The biodiesel samples were designated by adding the letter ″B″ as a prefix to the corresponding catalyst name. For example, the biodiesel sample produced using the catalyst OSW50_800_1 was named as B_OSW50_800_1.

To assess catalyst reusability, the OSW50_800_1 sample (which is the catalyst prepared in the condition considered optimal within the experimental domain, as discussed below) was tested in multiple consecutive reacion cycles. After each reaction, the catalyst was dried at 100 °C for 16 h and reused under the same conditions, without regeneration. To indicate the reuse cycle, the suffix ‘rXc’ was added to sample names, where ‘X’ represents the cycle number (e.g., OSW50_800_1_r1c indicates the recovered catalyst and B_OSW50_800_1_r1c indicates the corresponding biodiesel). As shown in Table 3, new biodiesel samples were obtained, maintaining the stoichiometric proportion of the reagents, but with masses three times greater than those used in the optimization stage.

### 2.5 Characterization of the Catalysts

The catalysts were characterized using several analytical techniques, including X-ray diffraction (XRD), scanning electron microscopy (SEM), thermogravimetry (TG), differential thermal analysis (DTA), Fourier transform infrared (FTIR) spectroscopy, and ^23^Na solid-state nuclear magnetic resonance (NMR) spectroscopy.

The XRD experiments were performed at room temperature on a Shimadzu XRD-6000 diffractometer, operating at 40 kV and 30 mA. The diffraction angle (2θ) was varied from 10° to 80° in 0.02° increments, using Cu–Kα radiation (λ = 1.5418 Å).

The SEM images were recorded on a JEOL JSM6610LV microscope, with an adjustable acceleration voltage between 300 V and 30 kV, resolution from 3.0 to 15 nm using a tungsten filament and magnification from 5 to 300.000×. Coupled to the microscope was a Bruker XFlash Detector 6|10 Energy Dispersive X-ray Detector (EDX) with an analysis area of 10 mm^2^ and energy resolution of 121 eV in MnKα, 38 eV in CKα and 47 eV in FKα (100.000 cps).

The FTIR spectra were recorded in transmittance mode in the spectral range from 650 to 4000 cm^–1^, using the attenuated total reflection (ATR) method, on a PerkinElmer Spectrum 400 FTIR/FT-NIR spectrometer, with accumulation of a total of 16 scans. The TG/DTA curves were recorded using a Shimadzu DTG-60 balance; the derivative TG (DTG) curves were obtained numerically from the TG data. The initial sample mass was set to 3.0 mg, with a heating rate of 10 °C/min, from room temperature up to 1000 °C, under an O_2_ flow of 50 mL/min. The sample masses were normalized after the water release stage (around 200 °C). To determine the percentage of residual material following the combustion of the carbon matrix, a temperature of 850 °C was used. The content of inorganic material deposited on the porous carbon matrix was estimated by subtracting the ash content of the CSAC sample, Figure S1, (determined in prior work to be 2.5 wt %)^29^ from the residual mass of the catalyst at 850 °C.

Given the presence of sodium in the prepared catalysts, solid-state ^23^Na NMR spectra were also recorded at room temperature on a Varian/Agilent VNMR 400 MHz spectrometer, under a 9.4 T magnetic field (corresponding to a ^23^Na NMR frequency of 105.8 MHz), using a triple resonance radiofrequency (RF) probe. Powder samples were packed into 4 mm diameter zirconia rotors for magic-angle spinning (MAS) experiments conducted at 14 kHz. Single-pulse excitation experiments were performed with a pulse duration of 1.0 μs, a repetition delay of 2.0 s, a spectral window of 50 kHz, and an acquisition time of 20.48 ms, with 64 accumulated transients. The spectra were obtained through Fourier transformation of the free induction decays (FIDs), with the frequency shifts (δ) referenced against an aqueous NaCl solution, using solid NaCl (δ = 7.2 ppm) as the external secondary reference.^37^

### 2.6 Characterization of Biodiesel Samples

The conversion of soybean oil into biodiesel in the transesterification reaction was quantified by solution ^1^H NMR spectroscopy, using the aforementioned NMR spectrometer, operating at a frequency of 399.7 MHz, at room temperature. Samples (approximately 5 mg) were dissolved in 600 μL of deuterated chloroform and placed in a glass tube with an outer diameter of 5 mm. The experiments were conducted using a 5.9 μs excitation pulse (π/4), with a spectral window of 6410.3 Hz, a repetition delay of 1.16 s, and an acquisition time of 3.83 s, with accumulation of 32 transients. The NMR spectra were obtained by Fourier transformation of the FIDs, with the chemical shifts referenced to tetramethylsilane (TMS) present in the solvent.

The biodiesel conversion was calculated according to eq 1, where I_CH_2__ is the integrated intensity of the triplet signal near 2.30 ppm due to the methylene group (αCH_2_) adjacent to the ester moiety of the triacylglycerol molecule and I_CH_3__ is the integrated intensity of the singlet signal near 3.66 ppm due to the methoxyl group (OCH_3_), which is characteristic of methyl esters.^38–41^

$$C_{\mathrm{NMR}}(\%)=\frac{2I_{{\mathrm{CH}}_{3}}}{3I_{{\mathrm{CH}}_{2}}}\times 100$$ 1

The ^1^H NMR spectra of the biodiesel samples had their intensities normalized in relation to the methylene group signal (around 2.30 ppm); the integrations used to calculate the spectral intensities were performed in the spectral intervals from 2.25 to 2.35 ppm (αCH_2_) and from 3.62 to 3.70 ppm (OCH_3_).

To determine the fatty acid profile of methyl esters in the biodiesel samples, gas chromatography–mass spectrometry (GC-MS) analyses were performed using a Shimadzu GCMS-QP2010 Ultra spectrometer equipped with a VB5 capillary column (30 m × 0.32 mm × 0.25 μm). These analyses were conducted with two representative samples (B_OSW50_800_1 and B_OSW20_600_1) selected among the biodiesel samples produced in this work. After purification with distilled water, the biodiesel sample was diluted to 5 mg/mL in dichloromethane, and a 1 μL aliquot was injected in split mode. The column oven was initially set at 80 °C for 1 min, then the temperature was increase to 200 °C at 6 °C/min, held for 2 min, and further increased to 320 °C at 10 °C/min, with a final hold time of 10 min. The injector was maintained at 290 °C, and the carrier gas flow rate was 1.5 mL/min.^42^

The leaching of inorganic species from the catalysts was investigated by determining the Ca, Mg and Na contents in the biodiesel samples produced in each reaction by flame atomic absorption spectrometry (F AAS), using the Analytik Jena AAS ZEEnit 700 instrument, following a previously established methodology.^43^

## 3 Results and Discussion

### 3.1 Optimization of Catalyst Preparation

Following the experiments detailed in Table 2, all biodiesel samples were quantified by ^1^H NMR, with C_NMR_ values obtained by integrating the signals at 2.30 and 3.66 ppm (eq 1). The resulting C_NMR_ values for each experiment are shown in Table 4. Effect values were derived from these measurements and variable significance was subsequently analyzed.

**Table 4:** Biodiesel Conversion Responses Obtained in the Screening Stage (Table 2) of the Preparation of the Catalysts

exp	biodiesel nomenclature	*C*_NMR_ (%)
1	B_OSW20_600_1	47
2	B_OSW50_600_1	69
3	B_OSW20_800_1	81
4	B_OSW50_800_1	85
5	B_OSW20_600_3	55
6	B_OSW50_600_3	65
7	B_OSW20_800_3	71
8	B_OSW50_800_3	71
9	B_OSW35_700_2	69
10	B_OSW35_700_2	62
11	B_OSW35_700_2	63

The Pareto chart shown in Figure S2 illustrates the significance of the variables, where the bar of the standardized effect that is beyond the red line is considered to indicate a significant variable. Therefore, analyzing Figure S2, it can be seen that the variable mass ratio (x_1_) and temperature (x_2_) were considered significant, with the temperature having a much larger influence on the C_NMR_ response. This variable also had a positive effect, indicating that the increase in the temperature used to prepare the catalysts is expected to lead to an increase in the C_NMR_ response. The variable x_3_ (residence time) was considered insignificant within the experimental domain. Based on these results, it can be concluded that the best experimental conditions for the preparation of the catalysts were: OSW/CSAC mass ratio of 50:20, heat treatment temperature of 800 °C, and residance time of 1 h. The choice of this set of experimental conditions is considered to represent the best efficiency in terms of C_NMR_ (85%), corresponding to the OSW50_800_1 catalyst.

### 3.2 Catalyst Characterization

Figure 1 shows the XRD patterns of the prepared catalysts. All the analyzed samples presented magnesium oxide (MgO, periclase) as one of the dominant crystalline phases, with the typical diffraction peaks observed at 2θ = 42.90, 62.29, and 78.60° (ICDD crystallographic data sheet #78–0430).^44^ The MgO phase was produced as a consequence of the heat treatment conducted for the catalyst production, due to the thermal decomposition of magnesium hydroxide (Mg­(OH)_2_, brucite),^45^ which was formed during the preparation of the catalyst precursors (Figure S3). The main diffraction peaks due to Mg­(OH)_2_ were observed at 2θ = 18.59, 38.02 and 50.86° (ICDD crystallographic data sheet #07–0239).^44^ When preparing a heterogeneous catalyst based on dolomite, Murguia et al.^26^ identified also MgO as one of the phases obtained after thermally treating a mixture of dolomite (precalcined at 900 °C for 4 h) with sodium nitrate at 900 °C for 4 h.

**Figure 1:**
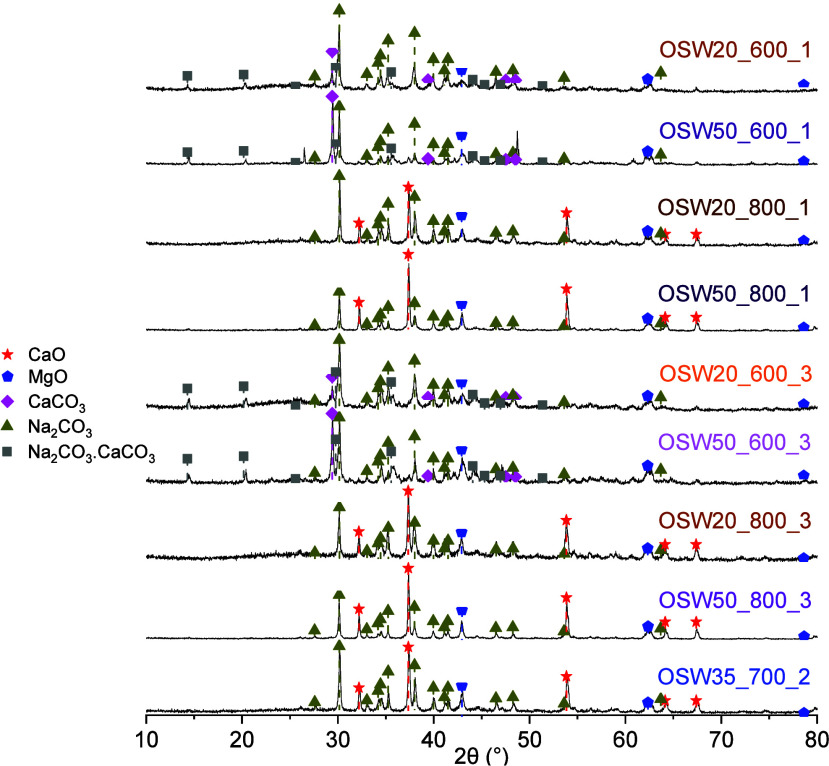
XRD patterns obtained for the prepared catalysts, with indication of the main identified phases.

Another phase observed in all catalysts was sodium carbonate (Na_2_CO_3_, natrite), with the main diffraction peaks at 2θ = 30.14, 35.23 and 38.00° (ICDD crystallographic data sheet #37–0451).^44^ This phase arose as a result of the reaction of NaOH with the carbon matrix (CSAC) during the production of the catalyst precursors,^7,46^ its formation being observed in the stage that preceded the heat treatment to obtain the catalysts (Figure S3).

The catalysts OSW20_600_1, OSW50_600_1, OSW20_600_3, and OSW50_600_3 (all heat-treated at 600 °C) also presented diffraction peaks corresponding to calcium carbonate (CaCO_3_, calcite), at 20 = 29.40, 39.41 and 48.50° (ICDD crystallographic data sheet #83–1762),^44^ and to the double carbonate Na_2_CO_3_.CaCO_3_ (nyerereite), at 20 = 20.16, 29.77 and 35.56° (ICDD crystallographic data sheet #28–1059).^44^

The CaCO_3_ phase identified in the catalysts may be attributed to an excess of OSW that did not fully react with NaOH during the preparation of the catalyst precursors, as indicated in Figure S3. Both the precursor samples OSW35 and OSW50 contained CaCO_3_ in their composition, supporting this hypothesis. Another possibility is that CaCO_3_ was formed during the heat treatment step. In fact, the precursor OSW20, with a smaller amount of OSW, showed initially no detectable CaCO_3_; this sample exhibitted Ca­(OH)_2_ (portlandite) as its dominant phase (Figure S3), with the main diffraction peaks at 2θ = 18.00, 34.10 and 47.12° (ICDD crystallographic data sheet #44–1481).^44^ It is likely that CaCO_3_ was formed in these cases as a consequence of the decomposition of Ca­(OH)_2_ in the presence of the carbon-rich support (CSAC).^7,46^ It is also worth noting that the double carbonate Na_2_CO_3_.CaCO_3_ was identified in the same samples containing CaCO_3_, indicating the reaction between Na- and Ca-containing species during the heat treaments; this phase is not stable at high temperatures, which explains the disapperance of the corresponding diffraction peaks for the samples prepared at 700 and 800 °C.,^7,35,36,47,48^

Therefore, the catalysts OSW35_700_2, OSW20_800_1, OSW50_800_1, OSW20_800_3 and OSW50_800_3 (prepared at 700 and 800 °C) no longer presented the double carbonate. The corresponding XRD patterns were dominated by the aforementioned MgO and Na_2_CO_3_ phases, besides the presence of well-defined diffraction peaks due to the CaO phase (peaks at 2θ = 32.20, 37.34 and 53.85° ICDD crystallographic data sheet #37–1497).^44^ The formation of CaO was clearly a consequence of the thermal decomposition of CaCO_3_, expected to occur above 600 °C.^30,49^

These results are consistent with previous findings reported by Faria et al.^35^ In their investigation on the optimization of catalyst preparation parameters using commercial CaO and activated carbon, those authors reported the formation of CaO, Na_2_CO_3_, CaCO_3_, and Na_2_CO_3_.CaCO_3_ crystalline phases, whose occurrence was strongly dependent on the heat treatment temperature used in the preparation of the catalysts.

To analyze the microstructure of the prepared catalysts’ surfaces, SEM images were recorded, as shown in Figure 2. The SEM images revealed the presence of many structures with different shapes (including needle-like crystals and nearly spherical particles) and with sizes in the μm scale, dispersed on the catalyst’ surfaces. As all prepared samples were rich in Na_2_CO_3_, it is likely that most of these elongated structures correspond to Na_2_CO_3_ crystals; in fact, the formation of needle-like crystals for this phase has been previously reported.^50^ The elemental maps obtained with EDX are shown in Figure S4 and Figure S5. These maps show the predominance of Na, C, and O in the elongated, needle-like structures (case of the OSW50_600_1 sample) and the concentration of Ca, Mg, and O in spheroidal particles (see, for example, the elemental maps obtained for the OSW50_800_1 sample). The occurrence of CaO particles is particularly evident in the EDX map of the OSW50_800_1 sample. The presence of the activated carbon matrix can also be clearly recognized in some of the images shown in Figure 2 (see, for example, the images obtained for the OSW20_600_3 and OSW50_600_3 samples). The deposition of the inorganic structures over the surface of the activated carbon matrix is more clearly observed in the SEM images recorded with lower magnification, shown in Figure S6. Together with the previously discussed XRD results, the SEM/EDX results show clearly that the prepared catalysts are composed of mixtures of inorganic phases deposited over the activated carbon support.

**Figure 2:**
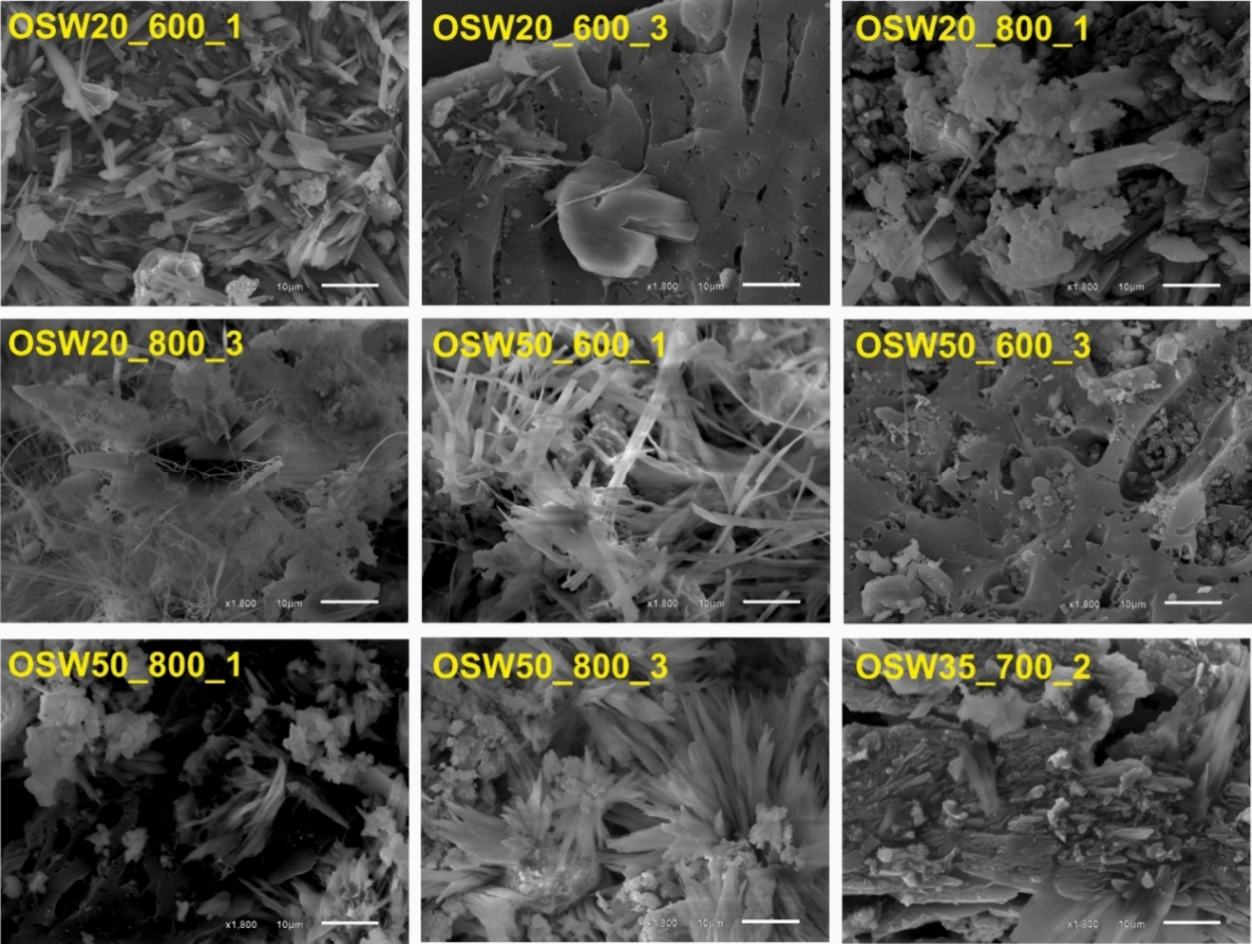
SEM images recorded at 1800× magnification for the prepared catalysts.

Among the phases present in the prepared catalysts, CaO is known to exhibit the highest catalytic activity in the transesterification reaction. As for the other identified phases, it is known that MgO, Na_2_CO_3_, and Na_2_CO_3_. CaCO_3_ also exhibit some catalytic activity, whereas this is negligible for CaCO_3_, considering the reaction conditions used in this work.^7,35,36,51–54^ It is thus not surprising that the catalysts that led to highest biodiesel conversions (see Table 4) were those prepared at 800 °C, which are rich in CaO and have no detected CaCO_3_. But it is also noteworthy that even the samples prepared at 600 °C (a temperature too low for the formation of CaO from the decomposition of CaCO_3_) exhibit fair catalytic activity (corresponding to C_NMR_ values roughly in the range 50–60%), which is mostly attributed to the presence of Na-containing carbonates deposited on the activated carbon matrix.

Figure 3 presents the FTIR spectra obtained for the prepared catalyts. The presence of the activated carbon matrix in the catalysts is evidenced by the bands in the region between 1910 and 2330 cm^–1^, also observed for CSAC and the catalyst precursors (Figure S7). In this spectral region, bands are prominent around 2000 and 2100 cm^–1^, indicating C – H bonding in aromatic compounds. The FTIR bands observed in this work for the CSAC sample are similar to the bands that Ho and Adnan^55^ attributed to activated charcoal prepared from coconut shells. Furthermore, it is important to note that the intensities of the bands in this spectral region varied according to the different weight fractions corresponding to the activated carbon support in each catalyst.

**Figure 3:**
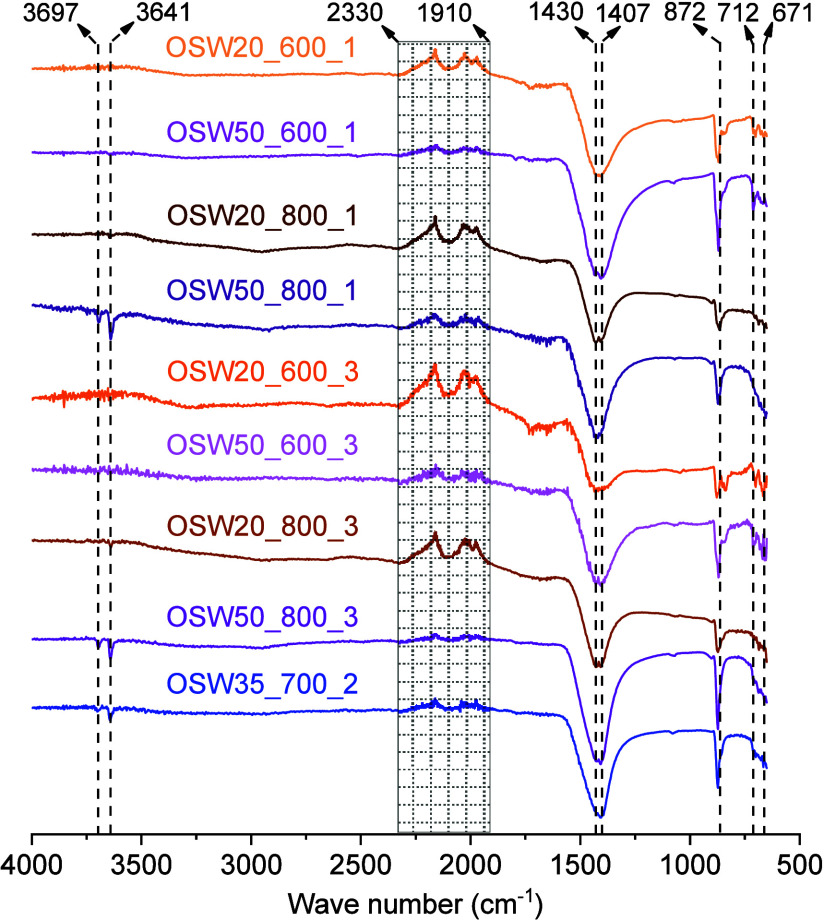
FTIR spectra obtained for the prepared catalysts.

Depending on the preparation conditions, the previously discussed XRD results (Figure 1) showed that the prepared catalysts contained three forms of carbonates: CaCO_3_, Na_2_CO_3_, and the double carbonate Na_2_CO_3_·CaCO_3_. The presence of the carbonate ion (CO_3_^2–^) was confirmed in the FTIR spectra through the detection of the vibrational modes at 872 and 1430 cm^–1^, corresponding to out-of-plane bending and doubly degenerate planar bending, respectively.^56^ These assignments are in line with the report by Widiarti et al.,^25^ who, in their study on catalyst preparation via coprecipitation and hydrothermal synthesis using dolomite as a precursor, attributed the bands observed at 875 cm^–1^ and 1421 cm^–1^ to the vibrational modes of the carbonate ion (CO_3_^2–^). Other bands due to Na_2_CO_3_, were identified at 671 and 700 cm^–1^,^57^ whereas the presence of the double carbonate (Na_2_CO_3_·CaCO_3_), was confirmed by the band at 712 cm^–1^.^58^

The CaO phase present in the catalysts heat-treated at 700 and 800 °C is capable of absorbing moisture and partially converting to Ca­(OH)_2_.^59,60^ The presence of the O – H bond in this compound is related to the band observed at 3697 cm^–1^ (also present in the FTIR spectra of the catalyst precursors, Figure S7).^61^ Similarly, Widiarti et al.^25^ observed a band at 3643 cm^–1^, attributed to the OH group of calcium hydroxide. The band at 3641 cm^–1^ is also attributed to O–H bond vibration in adsorbed water molecules.^56^ The peak at 671 cm^–1^ observed in the FTIR spectra is related to the stretching vibrations of the Mg – O bond,^62,63^ confirming the formation of MgO.

The TG curves recorded for the prepared catalysts are shown in Figure 4 and Figure 5. In all these curves, the residual weight values were normalized at 200 °C (i.e., after the release of moisture), so all the weight loss values were calculated on a dry basis. The corresponding DTA curves are shown in Figure S8. Since all the prepared catalysts were prepared using an activated carbon as support, a significant weight loss is observed in the temperature range from 300 to 450 °C in all TG curves, due to the burning of the carbon matrix.^64^ This event is also detected as a large exothermic peak in the DTA curves (Figure S8). After the carbon combustion, the catalysts prepared with increasing OSW/CSAC mass ratios presented progressively smaller residual masses (in the order OSW20 < OSW35 < OSW50), which is clearly an indication of the amount of inorganic phases in each set of samples. It is also possible to notice that the residual masses are influenced by the temperatures and times of thermal treatments that were used in the preparation of the catalysts (Table 2).

**Figure 4:**
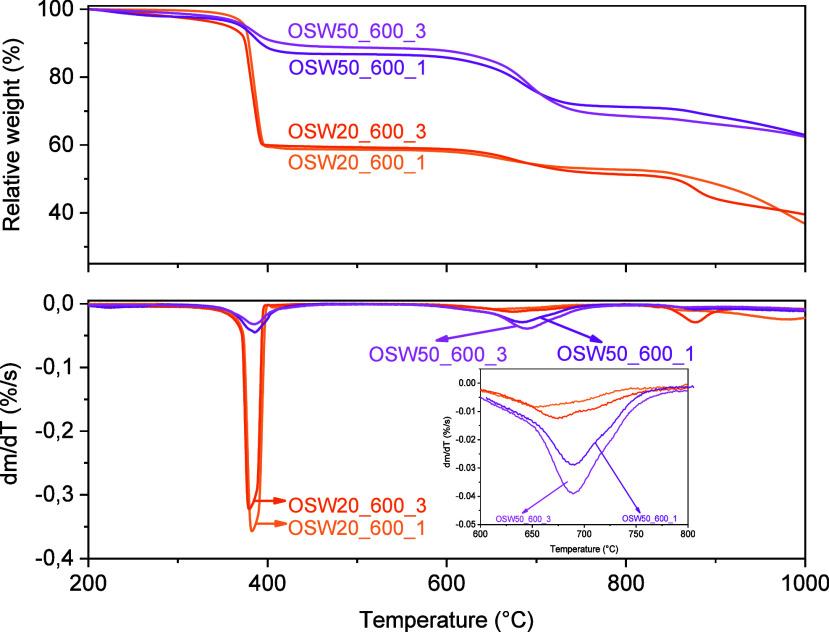
TG/DTG curves recorded under an oxidizing atmosphere for the catalysts prepared at 600 °C.

**Figure 5:**
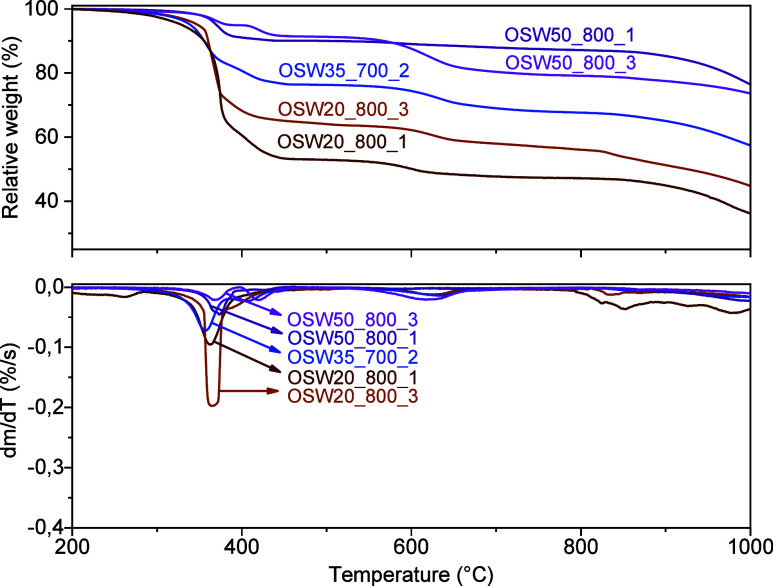
TG/DTG curves recorded under an oxidizing atmosphere for the catalysts prepared at 700 and 800 °C.

According to Figure 4 and Figure 5, the catalysts prepared at 600, 700, and 800 °C showed weight losses at similar temperatures. After the combustion of the carbon matrix, the TG curves exhibited in Figure 4 indicated a weight loss in the temperature range from 600 to 750 °C, attributed to the decomposition of Na_2_CO_3_.CaCO_3_ and CaCO_3_.^47,65^ In contrast, Na_2_CO_3_ remains thermally stable up to 850 °C;^7,35,66^ from this temperature onward, a slow weight loss was observed, suggesting the beginning of the decomposition of this compound.^66,67^

The weight losses observed in the TG curves obtained for the prepared catalysts are in line with the results reported by Faria et al.,^35^ who were able to identify similar weight losses related to the burning of the activated carbon used as a support, as well as the thermal decomposition of Na_2_CO_3_.CaCO_3_ and CaCO_3_. They also observed the start of thermal decomposition of Na_2_CO_3_ at 850 °C.

Solid-state ^23^Na NMR is a method extensively used for the study of Na-containing materials; with this technique, it is possible to detect contributions both from crystalline and from disordered environments, thus allowing the characterization of the chemical and structural features of the analyzed materials in a approach complementary to XRD (which is a technique appropriate for the characterization of crystalline phases).^37,68,69^ Figure 6 presents the solid-state ^23^Na NMR spectra obtained for the prepared catalysts and for a sample of pure Na_2_CO_3_ (included for comparison purposes). All the observed resonances were quite broad, a characteristic attributed to quadrupole coupling effects that are typically observed for nuclei with spin 3/2 (such as ^23^Na) located in sites with non-negligible electric field gradients (EFGs). These resonances corresponded in all cases to the central transition of the quadrupole-split energy levels of the ^23^Na nuclei interacting both with the external magnetic field of the NMR spectrometer and the internal EFG at each nuclear site.^32^

**Figure 6:**
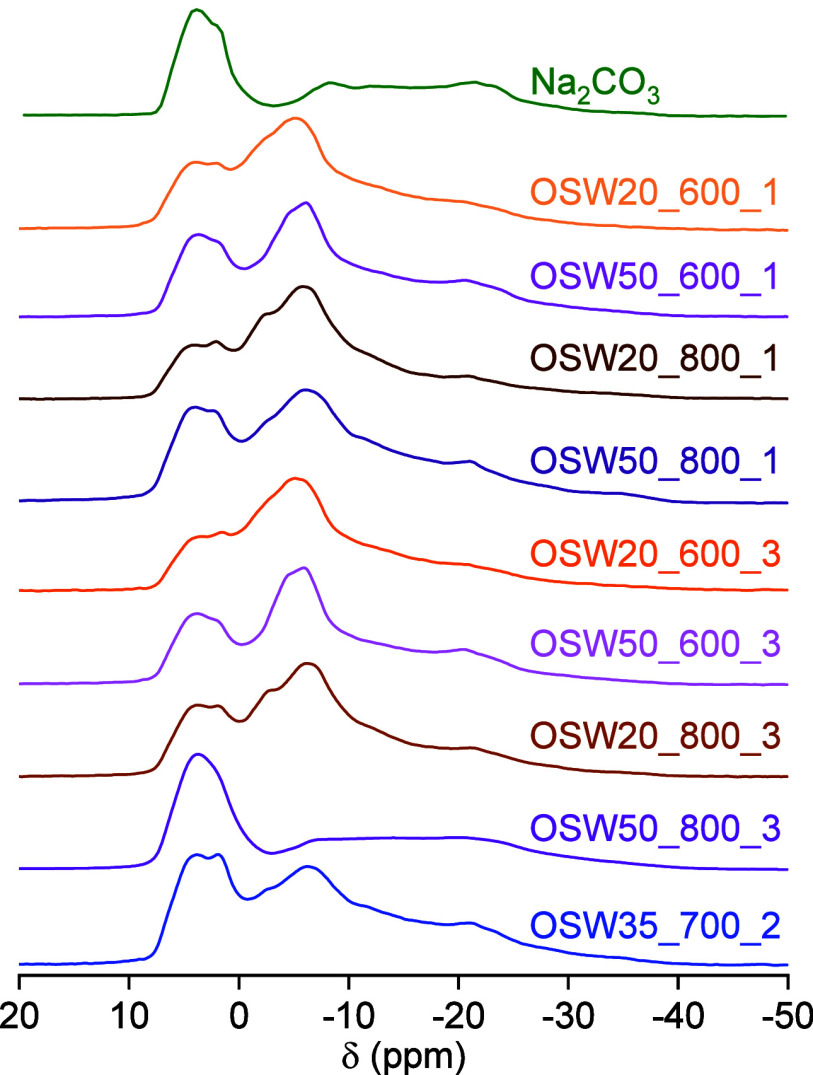
Solid-state ^23^Na NMR spectra recorded for the prepared catalysts and for a sample of pure Na_2_CO_3_.

The ^23^Na NMR spectrum obtained for pure Na_2_CO_3_ showed a resonance composed of spectral contributions components associated with the distinct crystallographic sites in the Na_2_CO_3_ structure, including an intense contribution between 0 and 10 ppm and a broader one in the range between −5 and −30 ppm.^35,46,70,71^ The ^23^Na NMR spectra obtained for all the prepared catalysts contained contributions resembling the ones observed for pure Na_2_CO_3_, confirming the dominant presence of this phase in all samples (in agreement with the XRD patterns of Figure 1). In the case of the OSW50_800_3 sample, the ^23^Na NMR spectrum was essentially identical to that of Na_2_CO_3_. On the other hand, other broad components were detected in the spectra obtained for most catalysts. For the samples prepared at 600 °C, the presence of the double carbonate Na_2_CO_3_·CaCO_3_ (identified by XRD) gave rise to a maximum around −5 ppm and a broad resonance roughly between −10 and −20 ppm (partially superimposed to the broad contribution due to Na_2_CO_3_).^35^ It is interesting to note that a similar spectral profile was observed also for the samples prepared at 700 and 800 °C (even if the crystalline phase of the double carbonate had not been identified by XRD in these samples). This suggests that broad resonances due to structurally disordered phases (which can include phases with severely reduced crystallite sizes and large surface/volume ratio) are also contributing to the ^23^Na NMR spectra of the catalysts. In fact, previous investigations dealing with Na-containing species in porous carbon materials have revealed the presence of broad resonances centered around −7 ppm in the ^23^Na NMR spectra, which were associated with Na^+^ ions in disordered environments.^7,29,35,69^ These structurally disordered Na-containing phases are expected to play a significant role on the catalytic activity of nanostructured materials,^35^ which evidence the relevance of their identification by solid-state ^23^Na NMR spectroscopy.

### 3.3 Catalytic Tests

The catalysts characterized in the previous section were used in the transesterification reaction of soybean oil and methanol to produce biodiesel. After catalyst separation, all reaction products exhibited a well-defined two-phase system corresponding to biodiesel and glycerol, as shown in Figure 7. This clear phase separation indicates the effectiveness of the transesterification reaction, confirming that soybean oil was successfully converted into biodiesel and glycerol using the catalysts listed in Table 2. The upper phase consists of a mixture of methyl esters (biodiesel), while the lower phase contains glycerol. These results are consistent with the solution ^1^H NMR results (see below), confirming the efficiency of the prepared catalysts for biodiesel production.

**Figure 7:**
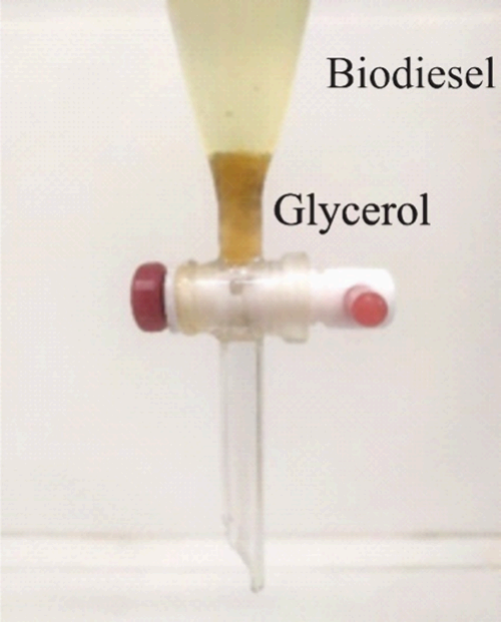
Illustration of the two-phase system obtained after separation of the catalyst in the case of the B_OSW20_800_1 sample.

Figure S9 shows the solution ^1^H NMR spectra recorded to monitor and quantify the conversion of soybean oil into biodiesel using the prepared catalysts (Table 2). As shown in Figure S9, all the obtained spectra showed a singlet at 3.66 ppm, indicating the formation of biodiesel in all cases; however, the relative intensity of this resonance relative to the triplet signal at 2.30 ppm due to the methylene group (taken here as reference) were markedly different. The spectra obtained for the biodiesel samples B_OSW20_600_1 and B_OSW50_800_1 showed the smallest and largest peaks at 3.66 ppm, respectively, which corresponded to the extreme values of biodiesel conversion (C_NMR_) values, informed in Table 4.

It is worth noting that, under the conditions used in the transesterification reactions in the present work, the CSAC sample does not show catalytic activity, as reported in previous work.^29^ Thus, the catalytic activity of the prepared catalysts is attributed to the phases obtained after the heat treatments at the temperatures and times indicated in Table 2. The largest C_NMR_ values were observed for the biodiesel samples B_OSW50_800_1 (85%) and B_OSW20_800_1 (81%). As shown in Figure 8, the biodiesel conversion showed a general trend of increasing as a function of the increase in the total content of inorganic phases deposited on the activated carbon support. This content was estimated by the difference between the residual mass observed in the TG curves recorded for the catalysts (Figure 4 and Figure 5) and that corresponding to the CSAC sample (Figure S1). This behavior is clearly a consequence of the catalytic activity of the inorganic phases identified in the XRD patterns (Figure 1). It is worth noting that the catalysts prepared at 800 °C were the ones presenting the highest C_NMR_ values, which is associated with the prevalence of CaO and Na_2_CO_3_ (which are known to be good catalysts for biodiesel production) in the composition of these samples.^7,51,52,72–74^

**Figure 8:**
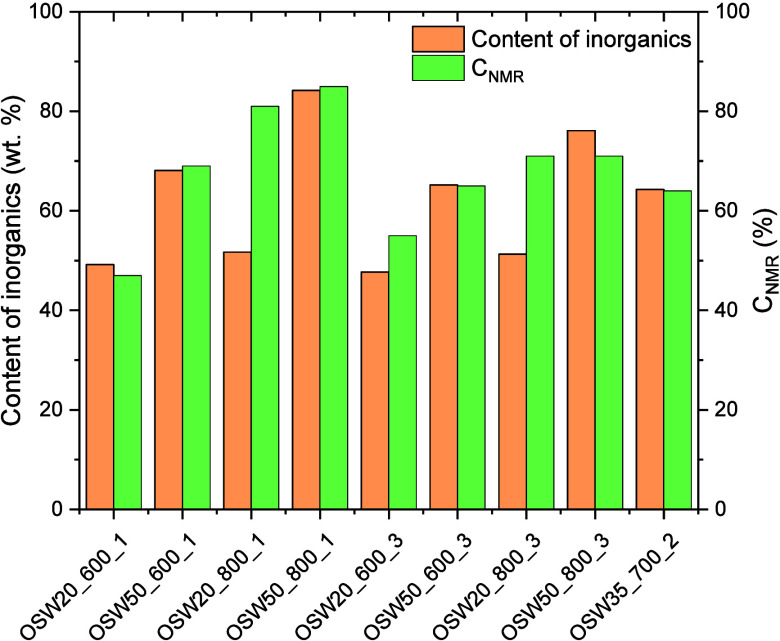
Comparison between biodiesel conversion values and the contents of inorganic phases deposited on the activated carbon support.

The profile of the methyl esters formed during the transesterification reaction to produce the biodiesel samples was analyzed using GC-MS on samples B_OSW50_800_1 and B_OSW20_600_1, as shown in Figure 9. Table S1 lists the primary chemical constituents of the biodiesel samples, along with their respective retention times and relative concentrations, determined by integrating the areas of the peaks in the chromatograms. The chromatograms in Figure 9 reveal that the most abundant methyl ester is methyl octadeca-9,12-dienoate, followed by methyl octadec-6-enoate and methyl hexadecanoate.^42,75^ In their study on biodiesel production using soybean oil as feedstock, Joshi et al.^75^ identified a predominance of methyl octadeca-9,12-dienoate ester. Such result is consistent with the findings observed in the present work. The total relative concentrations of the methyl esters exceeds 96.5% for all analyzed samples, thus meeting the European Standard EN-14214 compliance requirement.^30^ The biodiesel samples B_OSW50_800_1 and B_OSW20_600_1 exhibit relative concentrations values of unsaturated fatty acids of 78.61 and 82.23%, respectively. These percentages suggest that biodiesel B_OSW20_600_1 may have lower oxidative stability.The oxidative stability of a biodiesel sample affects fuel quality due to factors such as exposure to atmospheric oxygen or to high temperatures.^76^

**Figure 9:**
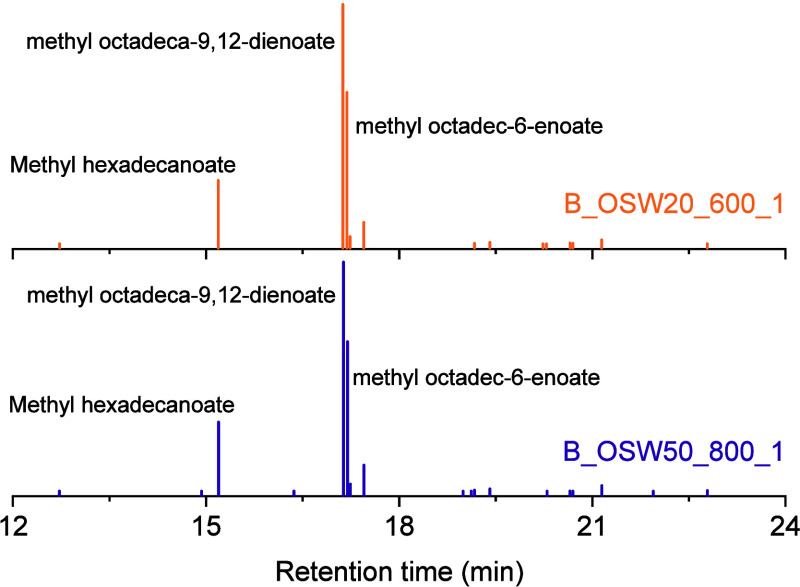
Gas chromatograms of biodiesel samples B_OSW50_800_1 and B_OSW20_600_1.

An important aspect that must be considered for biodiesel to be used on an industrial scale is the presence of metals such as Ca, Mg, and Na that could be leached into the reaction medium during biodiesel synthesis. The EN-14214 standard establishes that the combined levels of Ca and Mg cannot exceed 5.0 mg/kg; this limit is also considered for the combined levels of Na and K.^30^ Therefore, in order to check whether the biodiesel samples prepared using the catalysts described in this work adhere to the mentioned standard, the Ca, Mg, and Na contetns in the produced biodiesel samples were determined by F AAS analyses, as reported in Table 5.

**Table 5:** Metal Contents in the Prepared Biodiesel Samples Obtained by F AAS

sample	Ca (mg/kg)	Mg (mg/kg)	Na (mg/kg)
B_OSW20_600_1	1.4 (0.5)^a^ ^,^ ^b^	0.7 (0.0)^c^	<LQ_Na_ ^d^
B_OSW50_600_1	<LQ_Ca_	1.1 (0.0)	<LQ_Na_
B_OSW20_800_1	4.3 (0.1)	1.0 (0.0)	<LQ_Na_
B_OSW50_800_1	5.0 (0.1)	1.3 (0.1)	<LQ_Na_
B_OSW20_600_3	5.1 (0.2)	0.8 (0.0)	<LQ_Na_
B_OSW50_600_3	<LQ_Ca_	1.3 (0.2)	<LQ_Na_
B_OSW20_800_3	3.2 (0.2)	1.3 (0.1)	<LQ_Na_
B_OSW50_800_3	<LQ_Ca_	0.2 (0.0)	<LQ_Na_
B_ OSW35_700_2	12.5 (0.5)	5.2 (0.5)	<LQ_Na_

a The numbers in parentheses indicate the standard deviation of the measurements.

b Limit of quantification for Ca (LQ_Ca_) = 0.76 mg/kg.

c Limit of quantification for Mg (LQ_Mg_) = 0.15 mg/kg.

d Limit of quantification for Na (LQ_Na_) = 5.0 mg/kg.

These results show that all prepared biodiesel samples presented Na contents below the limit of quantification of the chosen protocol (by F AAS), which coincides with the upper limit established by the EN-14214 standard for the combined contents of Na and K.^30^ For most samples, the Mg contents were lower than the Ca contents, which can be attributed to the inferior amount of Mg present in the OSW material, as determined in a previous work.^29^

Regarding the combined Ca and Mg contents, Table 5 shows that most biodiesel samples exhibited values lower than or just slighly above the limit established by the EN-14214 standard. The two biodiesel samples corresponding to the highest C_NMR_ values in Table 4 (B_OSW20_800_1 and B_OSW50_800_1), for instance, had Ca + Mg contents of 5.3 and 6.3 mg/kg, respectively. Only for one sample (B_OSW35_700_2) the combined Ca + Mg content was expressively above that limit.

It is important to note, though, that all metal contents of the biodiesel samples produced using the carbon-supported catalysts prepared in this work were remarkably lower than the values corresponding to unsupported catalysts are used. In a recent study, Aleixo et al.^29^ reported the production of biodiesel from soybean oil and methanol using a CaO-rich unsupported catalyst prepared by heat treatment of OSW at 800 °C for 1 h; the Ca + Mg content of this biodiesel sample was 387 mg/kg. This value was significantly lower than the Ca content of 2274 mg/kg reported by Faria et al.^7^ when using commercial CaO as the catalyst of the same reaction between soybean oil and methanol to obtain biodiesel. This reduction in metal leaching highlights the effectiveness of OSW-based catalysts in minimizing contamination in biodiesel compared to commercial CaO. However, the Ca + Mg content remained approximately 77 times lower than the limit set by standard EN-14214. Notably, when OSW was used in the present work for the production of carbon-supported Na- and Ca-containing catalysts, the metal leaching was severely reduced, with the achievement of metal contents inferior or quite close to the regulatory limits.

For the optimized condition of catalyst preparation (sample OSW50_800_1), reuse tests were carried out while keeping the methanol-to-soybean oil mass ratio constant. The tests confirmed that up to two reuse cycles were viable, with a C_NMR_ value of 89% in the first cycle. In the second and third cycles, the C_NMR_ values were reduced to 77 and 11%, respectively. It is noteworthy that, in this study, the catalyst was reused without undergoing any regeneration treatment beyond simple recovery by centrifugation and drying in an oven. Although the number of reuse cycles was limited, this approach contrasts with other studies in the literature that report extended catalyst reuse through additional processing steps aimed at maintaining catalytic performance. For instance, Niu et al.^73^ achieved five reuse cycles using a cerium-doped dolomite catalyst, reaching an initial conversion of 97.21%; however, the catalyst was systematically washed with ethanol and dried prior to each reuse.

## 4 Conclusions

This study successfully employed factorial planning to optimize the synthesis of heterogeneous catalysts supported on activated carbon using OSW as a source of CaO and MgO. The chosen wet-mixing synthesis route enabled the formation of catalytically active inorganic phases (such as CaO, MgO, Na_2_CO_3_, Na_2_CO_3_·CaCO_3_, and CaCO_3_) deposited on the carbon support, with the heat treatment temperature identified as the most influential variable from the point of view of the catalyst performance. Catalysts prepared at 800 °C, rich in CaO, achieved the highest biodiesel conversions (∼80%), while those synthesized at 600 °C also showed satisfactory performance (conversions in the range 50–60%) due to the presence of Na-based carbonates. Beyond their catalytic efficiency, the materials demonstrated environmental and economic advantages, given their low metal leaching and the use of industrial residues, aligning with the EN-14214 standard and reinforcing the potential of this method for sustainable biodiesel production.

## Supplementary Material



## Notes

Additional results are presented in the following figures and tables: TG and DTG curves recorded in an oxidizing atmosphere for the CSAC sample (Figure S1); Pareto chart showing the significance of the effects calculated for the screening stage of the preparation of the catalysts (Figure S2); XRD patterns obtained for the catalyst precursors­(Figure S3); SEM image of the OSW50_600_1 sample (top/left) and the corresponding EDX elemental maps for the elements Ca, Mg, Na, C and O (Figure S4); SEM image of the OSW50_800_1 sample (top/left) and the corresponding EDX elemental maps for the elements Ca, Mg, Na, C and O (Figure S5); SEM images recorded at 200× magnification for the prepared catalysts (Figure S6); FTIR spectra obtained for the indicated samples (Figure S7); DTA curves recorded under an oxidizing atmosphere for the indicated catalysts (Figure S8); ^1^H NMR spectra of the biodiesel samples obtained using the prepared catalysts, with indication of the corresponding biodiesel conversion values (Figure S9); Chemical constituents of the biodiesel samples: B_OSW50_800_1 and B_OSW20_600_1 (Table S1) (PDF)
