# Supplementary material for: Optimization of Catalyst Synthesis Using Ornamental Stone Waste Supported on Activated Carbon for Enhancing Biodiesel Production Efficiency
Source: ACS Omega. 2025 Jul 15;10(29):31536–48. doi: 10.1021/acsomega.5c02065 (PMC12311732; doi:10.1021/acsomega.5c02065)
Supplement: Supplementary file 1 [file ao5c02065_si_001.pdf]

Optimization of Catalyst Synthesis Using Ornamental Stone Waste Supported on Activated Carbon for Enhancing Biodiesel Production Efficiency

Fábio C. Aleixo[a,*], Diêgo N. Faria[a,b], Joycel V. Fernández[a], Daniel F. Cipriano[a], José G. A. Rodrigues[a], Gilberto M. Brito[a,c], Miguel A. Schettino, Jr.[a], Amanda Bolsoni[d], Geisamanda P. Brandão[d], Leonardo L. L. Silveira[e], Jair C. C. Freitas[a]

[a]Laboratory of Carbon and Ceramic Materials, Department of Physics, Federal University of Espírito Santo, Vitória, Espírito Santo 29075-910, Brazil.

[b]Laboratory of Chemical Sciences, State University of the Northern Rio de Janeiro, Campos dos Goytacazes, Rio de Janeiro 28013-602, Brazil.

[c]Engineering Unity, FAESA University Center, Vitória, Espírito Santo 29053-360, Brazil.

[d]Atomic Spectrometry Laboratory, Department of Chemistry, Federal University of Espírito Santo, Vitória, Espírito Santo 29075-910, Brazil.

[e]Center for Mineral Technology, Cachoeiro de Itapemirim, Espírito Santo 29300-970, Brazil.

*Email: fabio.aleixo@edu.ufes.br

Supporting Information

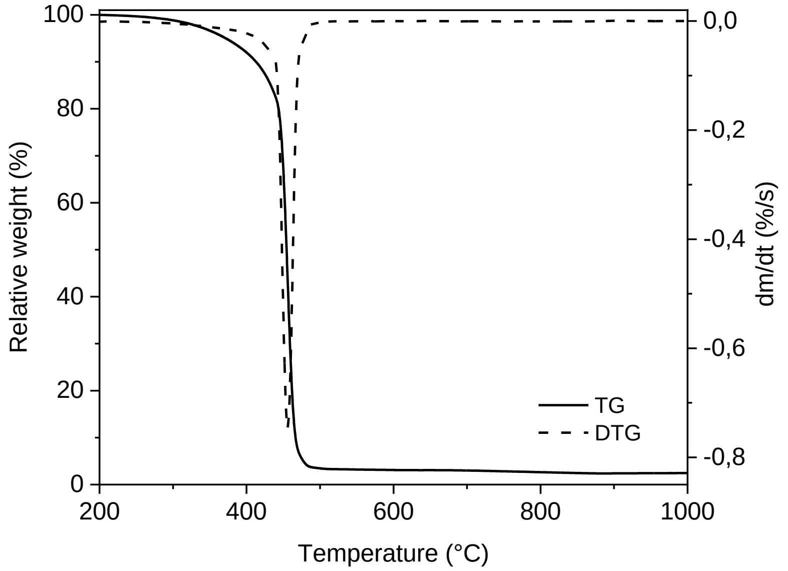

Figure S1– TG and DTG curves recorded in an oxidizing atmosphere for the CSAC sample.

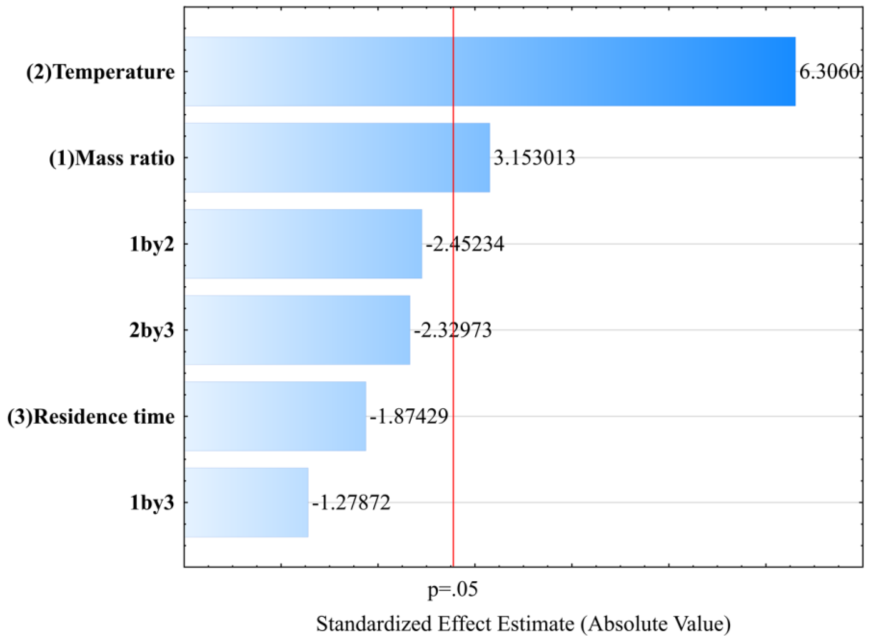

Figure S2– Pareto chart showing the significance of the effects calculated for the screening stage of the preparation of the catalysts.

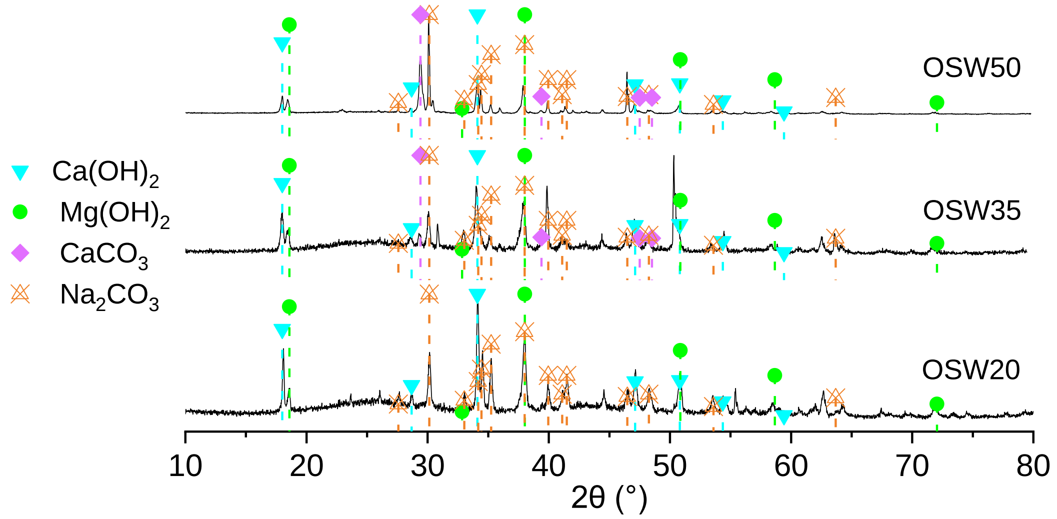

Figure S3– XRD patterns obtained for the catalyst precursors.

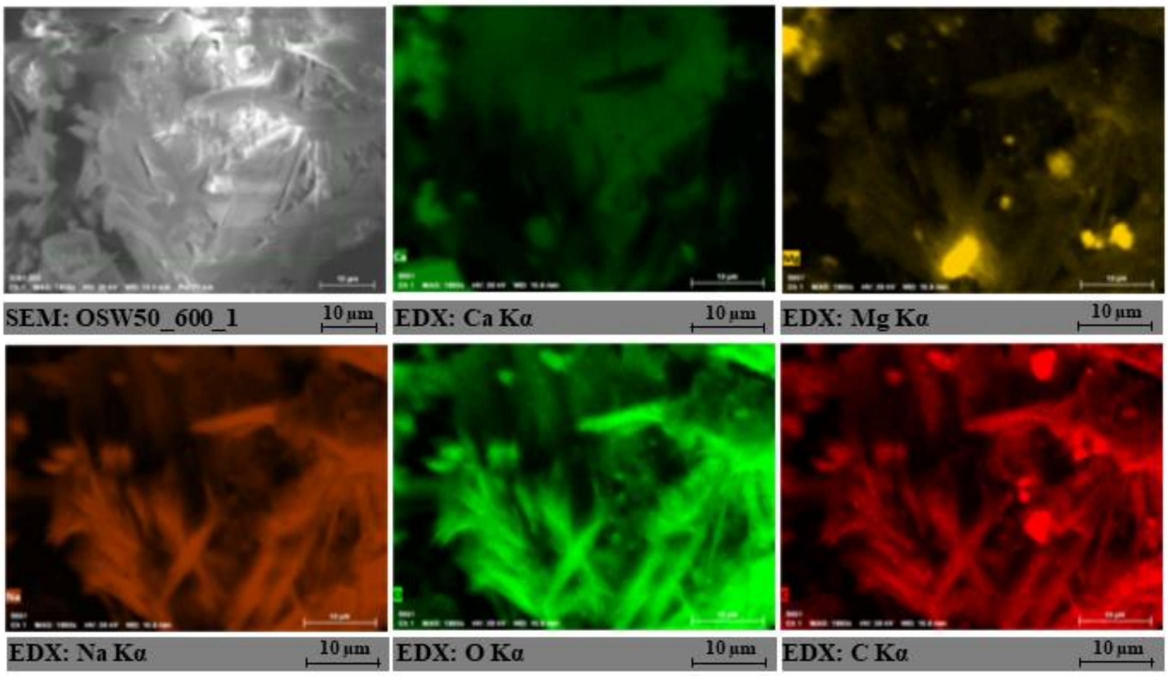

Figure S4– SEM image of the OSW50_600_1 sample (top/left) and the corresponding EDX elemental maps for the elements Ca, Mg, Na, C and O.

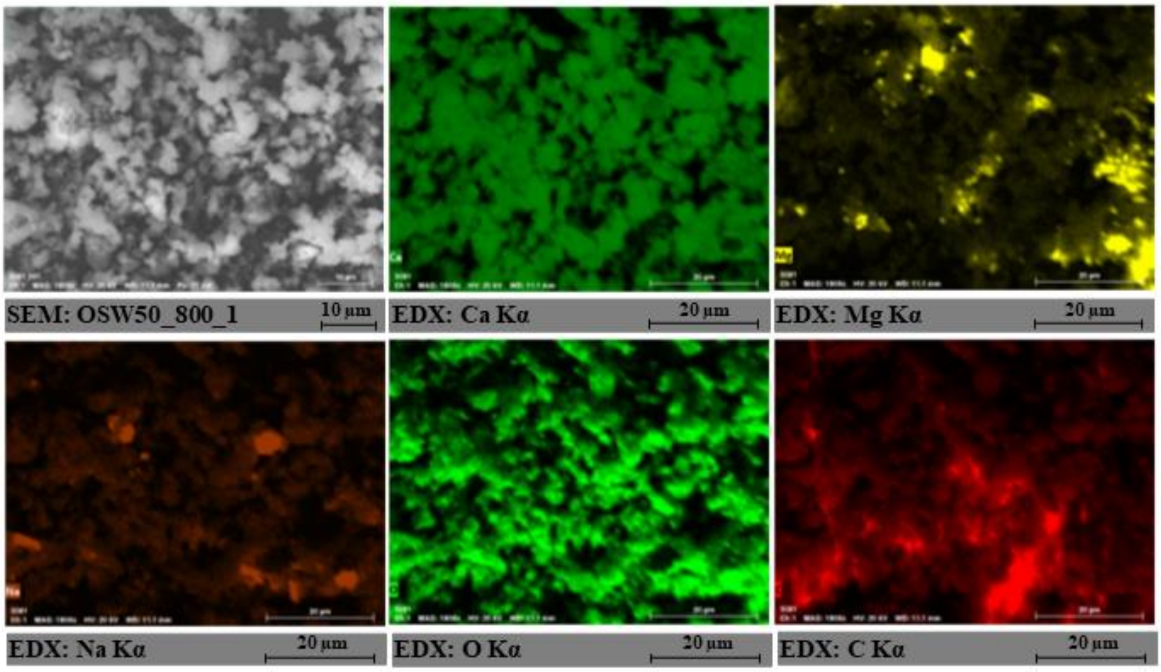

Figure S5– SEM image of the OSW50_800_1 sample (top/left) and the corresponding EDX elemental maps for the elements Ca, Mg, Na, C and O.

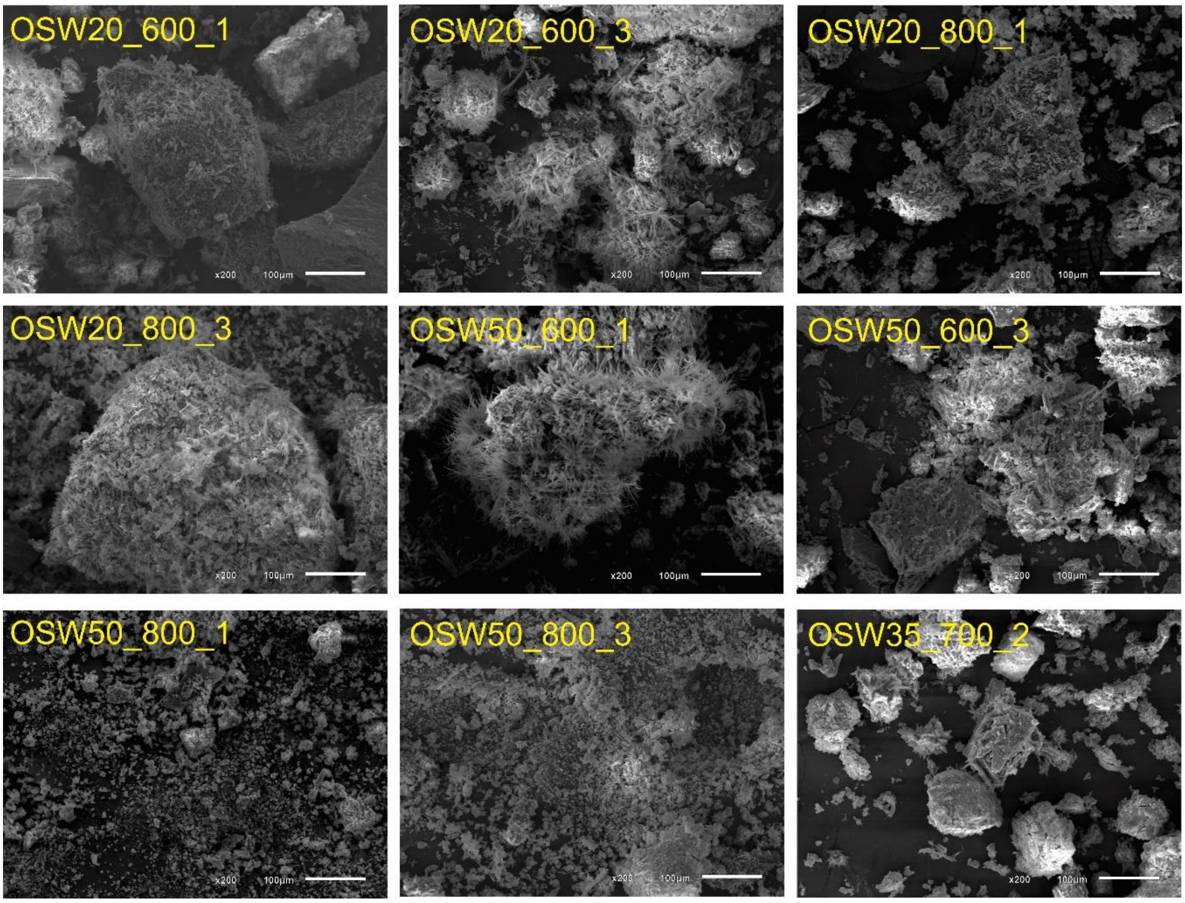

Figure S6– SEM images recorded at 200× magnification for the prepared catalysts.

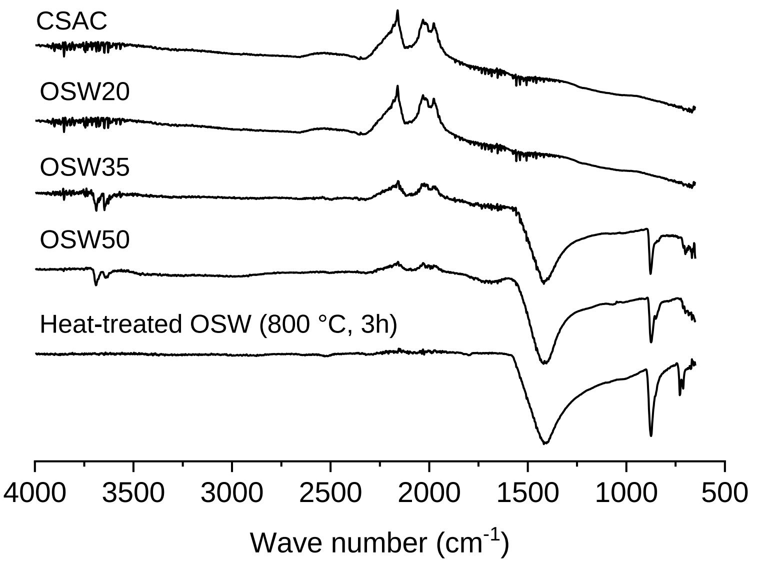

Figure S7– FTIR spectra obtained for the indicated samples.

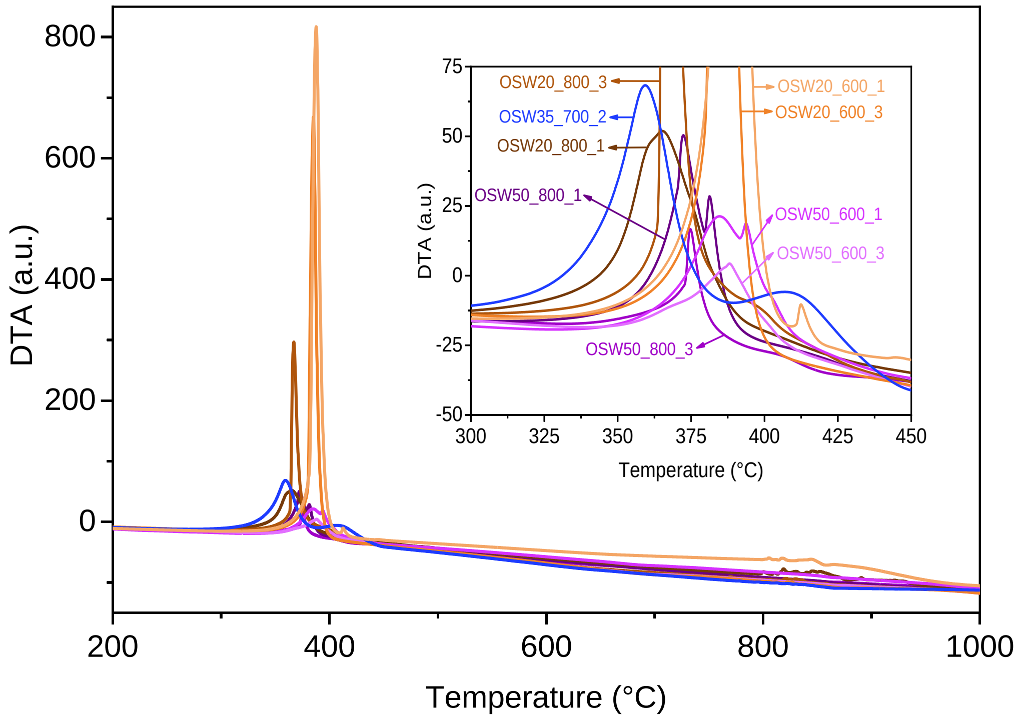

Figure S8– DTA curves recorded under an oxidizing atmosphere for the indicated catalysts.

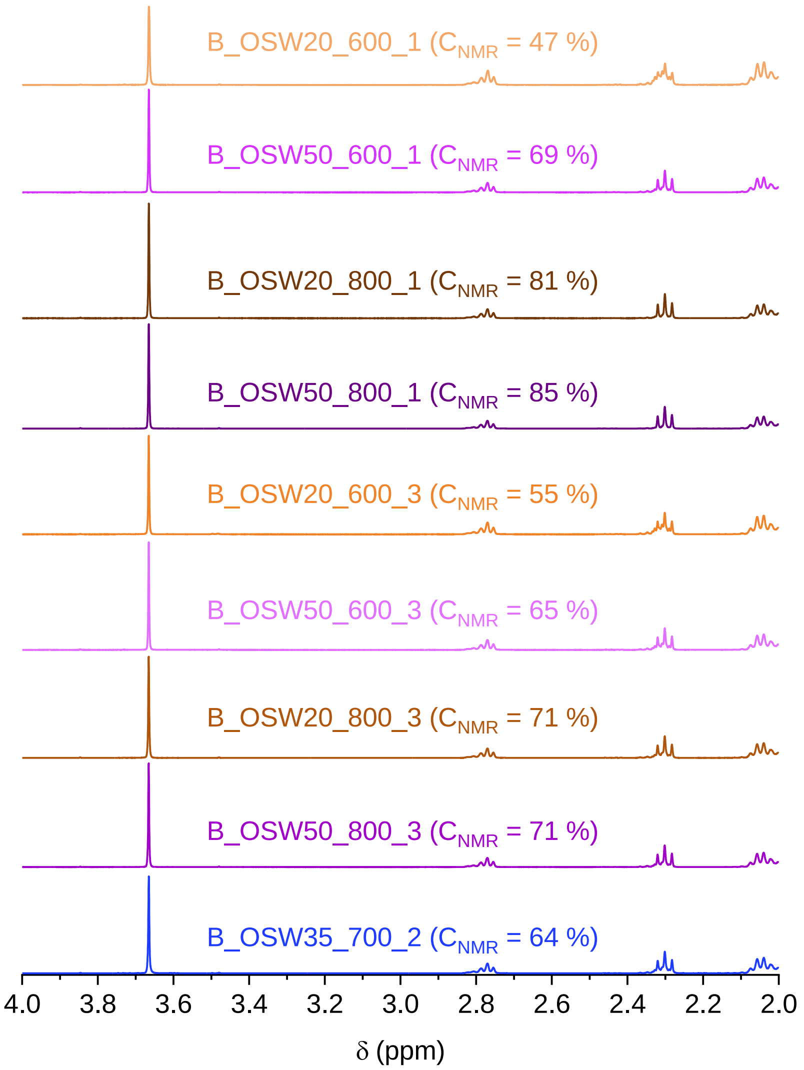

Figure S9– ^{1}H NMR spectra of the biodiesel samples obtained using the prepared catalysts, with indication of the corresponding biodiesel conversion values.

Table S1– Chemical constituents of the biodiesel samples: B_OSW50_800_1 and B_OSW20_600_1.

	**Chemical constituent (IUPAC name)**	**R_T (min)**	**B_OSW50_800_1 Rc (%)**	**B_OSW20_600_1 Rc (%)**
**1**	**Methyl tetradecanoate**	12,723	0.08	0.08
**2**	**Methyl (Z)-octadec-9-enoate**	14,931	0.05	-
**3**	**Methyl hexadecanoate**	15.195	14.03	12.89
**4**	**Methyl 15-methylhexadecanoate**	16,365	0.07	-
**5**	**Methyl octadeca-9,12-dienoate**	17.134	46.41	48.52
**6**	**Methyl octadec-6-enoate**	17.197	30.28	30.67
**7**	**Methyl (Z)-9-octadecenoate**	17,238	-	1.51
**8**	**Methyl octadec-9-enoate**	17,241	1.47	-
**9**	**Methyl octadecanoate**	17,452	5.31	4.38
**10**	**1,3-dihydroxypropan-2-yl (9Z,12Z,15 Z)- Octadeca-9,12,15-trienoate**	18,993	0.04	-
**11**	**Cycloheptadec-9-en-1-ol**	19.118	0.04	-
**12**	**Methyl (Z)-icos-11-enoate**	19.169	0.26	0.17
**13**	**Methyl 18-methylnonadecanoate**	19,409	0.48	0.34
**14**	**Methyl heneicosanoate**	20,297	0.03	-
**17**	**Tetradeca-9,12-dienyl acetate**	20,232	-	0.05
**18**	**Methyl (E)-octadec-9-enoate**	20,287	-	0.05
**19**	**(14R *)*-14-methylhexadec-8-yn-1-ol**	20,651	0.06	-
**20**	**(6Z,9Z)-pentadeca-6,9-dien-1-ol**	20,654	-	0.22
**21**	**Di-(9- octadecenoyl)- glycerol**	20,698	0.03	-
**22**	**2,3-bis[[(E)-octadec-9- enoyloxy] propyl (E)-octadec-9-enoate**	20,698	-	0.17
**24**	**Diphenylphosphorylbenzene**	21,450	-	0.87
**25**	**Methyl tetradecanoate**	21,146	1.16	-
**26**	**Methyl triacontanoate**	21,947	0.05	-
**27**	**Methyl tetracosanoate**	22,788	0.15	0.08
	**∑ Saturated compounds**		21.39	17.77
	**∑ Unsaturated compounds**		78.61	82.23
	**∑ Methyl esters**		97.5	96.62
